# Conservation planning integrating natural disturbances: Estimating minimum reserve sizes for an insect disturbance in the boreal forest of eastern Canada

**DOI:** 10.1371/journal.pone.0268236

**Published:** 2022-05-09

**Authors:** Marc Edwards, Kim Lisgo, Shawn Leroux, Meg Krawchuk, Steve Cumming, Fiona Schmiegelow

**Affiliations:** 1 Department of Renewable Resources, University of Alberta, Edmonton, AB, Canada; 2 Department of Biology, Memorial University of Newfoundland, St John’s, NL, Canada; 3 Department of Forest Ecosystems and Society, Oregon State University, Corvallis, OR, United States of America; 4 Département des sciences du bois et de la forêt, Université Laval, QC, Canada; University of British Columbia, CANADA

## Abstract

Large natural disturbances such as insect outbreaks and fire are important processes for biodiversity in forest landscapes. However, few methods exist for incorporating natural disturbances into conservation planning. Intact forest landscapes, such as in the North American boreal forest, can produce large natural disturbance footprints. They also have the potential to support large reserves but size estimates based on natural disturbance are needed to guide reserve design. Historical fire data have been used to estimate minimum dynamic reserves, reserve size estimates based on maintaining natural disturbance dynamics and ensuring resilience to large natural disturbance events. While this has been a significant step towards incorporating natural disturbance into reserve design, managers currently lack guidance on how to apply these concepts in areas where fire is not the dominant natural disturbance. We generalize the minimum dynamic reserve framework to accommodate insect outbreaks and demonstrate the framework in a case study for eastern spruce budworm (*Choristoneura fumiferana*) in the Canadian boreal forest. Our methods use geospatial analysis to identify minimum dynamic reserves based on a set of spatially explicit initial conditions, and simulation models to test for the maintenance of a set of dynamic conditions over time. We found considerable variability in minimum dynamic reserve size depending on the size of historic budworm disturbance events and the spatial patterns of disturbance-prone vegetation types. The minimum dynamic reserve framework provides an approach for incorporating wide-ranging natural disturbances into biodiversity conservation plans for both pro-active planning in intact landscapes, and reactive planning in more developed regions.

## Introduction

Large-scale natural disturbances such as insect outbreaks, fires and floods are among the essential processes shaping biodiversity [1]. However, many conservation plans do not include these processes, to the detriment of long-term biodiversity goals [2, 3]. Progress has been made towards incorporating natural disturbances into reserve design by combining site-selection algorithms with successional models [4–6] or predictions of disturbance risk [7]. Planners working in regions with active natural disturbance regimes would also benefit from quantitative estimates of the reserve size required to accommodate these events so as to maintain representative biodiversity over the long term [8–10]. Given the growing recognition of the importance of disturbance to ecosystems, and the likelihood for increased footprints of fire and insect outbreaks in forests globally [11, 12], integration of natural disturbance regimes explicitly into conservation planning initiatives is critical for global biodiversity conservation.

Natural disturbances operate at a range of spatial scales [13], and some disturbance types have the potential to produce large disturbance events that can affect all or much of a given reserve. Failure to account for these processes may undermine conservation goals associated with the designation of reserves. For example, Leroux et al. [4] demonstrated that reserve networks identified in a boreal ecosystem using conventional, static reserve design methods may not maintain their targets for fire-sensitive habitat and species at risk under the historical fire disturbance regime. Across the boreal region of Canada, natural disturbances affect large areas and play a significant role in shaping landscape structure as well as interacting with the life histories of many organisms [14–17]. The *de facto* protection of many boreal regions due to their inaccessibility [18] has facilitated the maintenance of natural disturbance regimes and biodiversity, but with increasing development, explicit plans are needed to conserve these areas and their constituent processes [19–21].

The size of disturbance events is an important factor in conservation planning, especially in areas where disturbance severity is high and large disturbances may exceed the size of existing reserves [22]. Minimum reserve areas have been suggested based on multipliers (e.g. one to two orders of magnitude) of the average historical disturbance size [23–25]. However, this does not account for the occurrence of exceptionally large disturbance events that have the potential to impact entire reserves [22]. The simplest way to design effective reserves in many regions may be to make reserves large relative to the maximum disturbance size [22]. Reserves designed in this way are more likely to accommodate the disturbance regime over time, and should therefore represent the patterns of biological organization resulting from landscape scale disturbance processes [22]. The emerging concept of ecological benchmarks fits well with this conceptual model. Ecological benchmarks are large, intact reserves designed to serve as reference sites for evaluating management actions elsewhere. They should maintain ecological processes over time, including natural disturbance-succession cycles, hydrological processes, nutrient cycles, and species interactions [26–28].

To effectively incorporate natural disturbance into reserve design, a methodological framework is needed to estimate minimum reserve sizes using historical, contemporary, and (ideally) projected future disturbance data. Based on the work of Pickett and Thompson [8], Leroux et al. [10] developed and applied methods to identify minimum dynamic reserves (MDRs) using recent historical records of wildfire size in the Canadian boreal forest. The MDR is an estimate of the reserve size required to support ongoing representation of fire-sensitive vegetation types in regions prone to large, high-severity wildfires. While wildfire is the dominant disturbance across much of the Canadian boreal region, multiple overlapping disturbances and disturbance probabilities occur, especially in regions with long fire return intervals where insect outbreaks are common [16]. Overlapping disturbances can interact such that the occurrence, extent or severity of a second disturbance is altered by the legacies of a first (i.e. linked disturbances [29]), or through compounding effects [30] that impact forest resilience [31, 32], potentially leading to unexpected and abrupt changes in ecological systems [33]. Planning methods such as the MDR are therefore needed for non-fire disturbance types, and ultimately for all combined and interacting disturbances in the boreal forest. In this paper, we extend the MDR framework to landscapes where defoliating insects play an important role in shaping forest composition [16]. Our objectives are as follows:

1. Develop a generalized framework that can guide calculation of MDRs for defoliating insects;
2. Apply the framework to the eastern spruce budworm (*Choristoneura fumiferana*), an important natural disturbance agent in Canada’s eastern boreal forests;
3. Demonstrate the application of these methods in conservation planning.

We consider this an important step towards a full-landscape model incorporating all disturbances and their interactions. These methods may be useful for pro-active conservation planning in intact regions still shaped by large-scale natural disturbances. They could also provide valuable information for reactive conservation and restoration in areas of biodiversity concern.

## Methods

### Generalized MDR framework for defoliating insect disturbance

Minimum Dynamic Reserves (MDRs) are the minimum reserve area required to incorporate natural disturbance and maintain ecological processes [10]. MDRs operationalize Pickett and Thompson’s [8] minimum dynamic area (MDA) concept that proposed reserve design, in the presence of natural disturbance, be based on the minimum area required to maintain the internal dynamics of vulnerable habitats as colonization sources to minimize the risk of extinction within the reserve. Leroux et al. [10] designed MDRs to be larger than all individual fire events, and to maintain minimum representation of fire-prone communities through time. They used a simulation framework of combined wildfire disturbance and vegetation dynamics to estimate an MDR size for their northern boreal study region.

Fire and insect disturbances are distinct ecological processes with different temporal and spatial properties, and different ecological impacts [16]. Fire events can be stand replacing under extreme conditions, typically change forest composition post-disturbance, and typically occur within a single fire season. In comparison, insect defoliators are host specific and outbreaks tend to result in cumulative damage over multiple years, with outbreak synchrony over wide areas, complex defoliation patterns, and high heterogeneity in canopy openness [34]. The host specificity of insect disturbances mean that these landscapes tend to have patchier patterns of mortality and age class distributions than after fire [35]. During spruce budworm outbreaks in eastern Canada, balsam fir trees are more vulnerable to mortality in dense, mature stands [36], and mature balsam fir (*Abies balsamea*) trees killed by the disturbance are often succeeded by their own regenerating understory cohort [37–39]. Younger stands and those with greater hardwood content will more often avoid mortality [36, 40]. A major effect of the disturbance is therefore a change in age structure, but not canopy composition as often observed following fire. Stand age is an important forest component for biodiversity in boreal ecosystems and forests with complex structure associated with gap dynamics can be reached after just 70 years [16, 24]. These fundamental differences in disturbance properties mean that the objectives of maintaining community composition and external seed sources are less relevant in insect dominated systems. A major requirement of our insect MDR method is therefore to maintain variation in host species age classes, a specific goal outlined in the original MDA criteria [8].

Building on the fire method of Leroux et al. [10], we make a number of changes to account for the differences between fire and insect disturbance. We require that an insect MDR be spatially contiguous, at least as large as the largest insect disturbance event, and that minimum amounts of host species age class groups be maintained within the reserve at all times. We apply a generalized MDR framework to identify insect-based MDRs using the following steps:

1) Vegetation dynamics: Define disturbance-prone vegetation types and develop a model of post-disturbance succession.
2) Data: Collate a sample of disturbance events with their attributes (e.g., size and severity) and a map of the initial vegetation state (vegetation types and age) for the planning region.

We then determine MDR size by a two-stage process:

3) Initial and dynamic conditions: Initial conditions are defined by the characteristics of the natural disturbance regime and vegetation properties of the planning region. These conditions dictate the minimum size and vegetation properties (type and area) that must be initially met by a candidate MDR. Dynamic conditions are defined as the minimum amount of each vegetation type to be maintained by an MDR at all times for a specified period (e.g., minimum 1 km^2^ over 250 years). Together these conditions increase the effectiveness of the MDR at maintaining the ecological processes associated with the natural disturbance regime over time.
4) Iterative MDR search: Identify candidate MDRs within the planning region that satisfy the initial conditions. More than one candidate MDR may be identified. Assess if any candidate MDRs satisfy the dynamic conditions using a simulation model. If all candidate MDRs fail to satisfy dynamic conditions, increment the MDR size and retest. The smallest candidate MDR that meets both initial and dynamic conditions is the estimated MDR size for the planning region.

### Case study–application of the generalized MDR framework

We illustrate this framework using a case study for spruce budworm on the island of Newfoundland, Canada. We selected this region because spruce budworm is the primary natural disturbance [41], and for the availability of historic disturbance and vegetation data. This region also has very low fire occurrence, and therefore fewer interactions between fire and insect disturbances, providing a simpler system in which to test our method.

To illustrate the application of this general MDR framework to regional conservation planning, we identify MDRs based on spruce budworm for a suite of ecoregions in the case study region and then evaluate the MDR properties of existing protected areas. This is not an exhaustive evaluation of the existing network. Because data are only available for one previous outbreak, the results carry high uncertainty. Our intent is not to make conservation recommendations based on the available data. Rather, it is to introduce the concepts and propose a method that we hope can be a first step towards a full-landscape approach to planning for natural disturbance in boreal regions.

#### Study area

**Fig 1:**
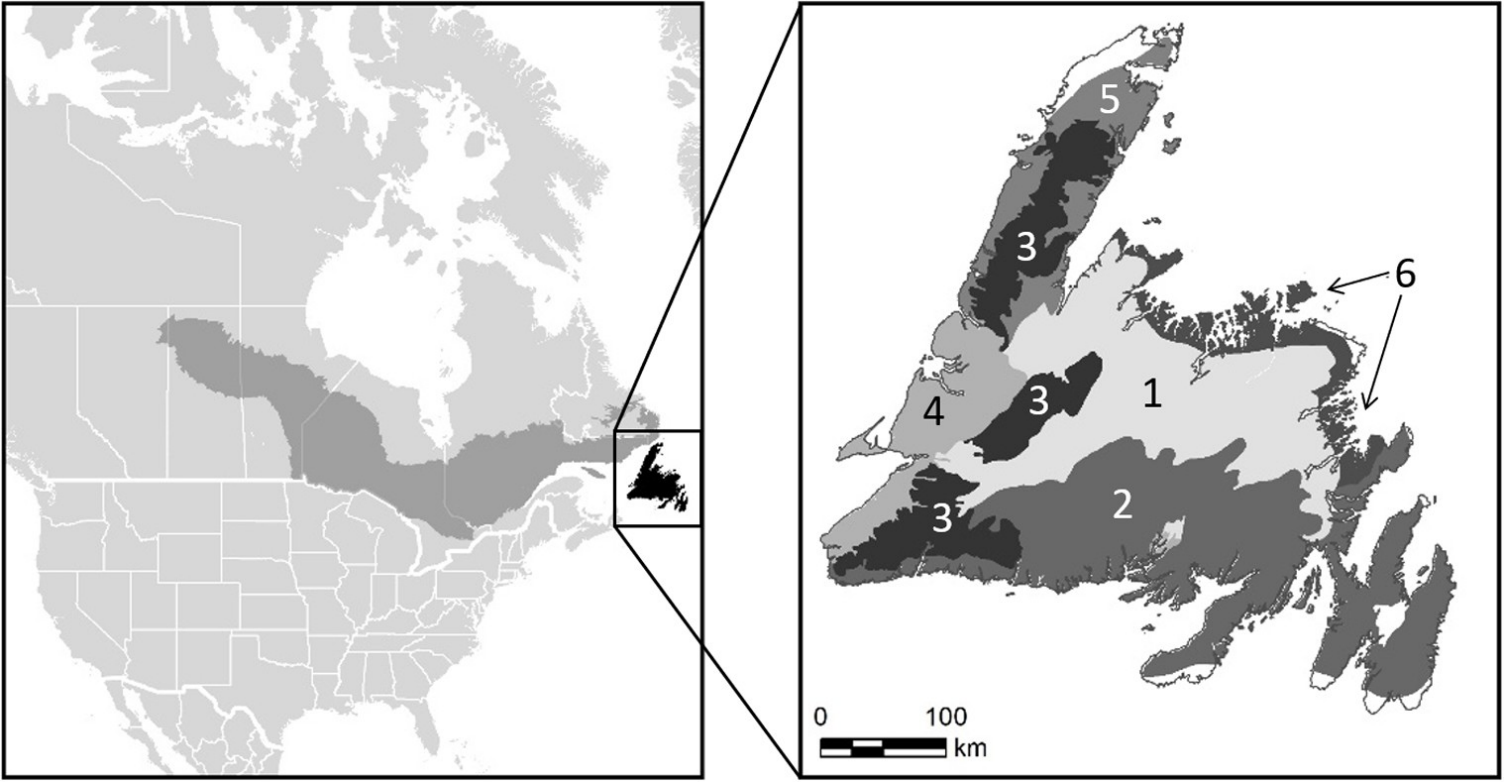
Study region map. Extent of the Boreal Shield ecozone in Canada and the locations of ecoregions 1 to 6 in our case study region.

Our study area is comprised of six ecoregions in the boreal shield ecozone in Newfoundland, Canada (Fig 1). These ecoregions were chosen based on the availability of spruce budworm disturbance data. Forest composition is dominated by balsam fir and black spruce (*Picea mariana*) which are the leading species in 55% and 39% of the forested area respectively [42]. White birch (*Betula papyrifera*) is the most common deciduous species and is the leading species in 5% of the forested area [42]. Spruce budworm is the most widespread natural disturbance, followed by hemlock looper (*Lambdina fiscellaria*) and wildfire. Forest harvesting is the most widespread human disturbance [41].

#### Spruce budworm

Eastern spruce budworm defoliation is a major disturbance in the boreal forest, especially in regions with relatively low fire activity [16]. The eastern spruce budworm feeds primarily on balsam fir, with white spruce (*Picea glauca*), red spruce (*Picea rubens*) and black spruce as secondary hosts experiencing lesser levels of defoliation [43]. Spruce budworm outbreaks in eastern Canada can last from 1–20 years and occur with an approximate return interval of 30–40 years [44, 45]. Tree mortality occurs after multiple years of defoliation, ranging from 3 to 8 years in eastern Canada [36, 46–48]. Spruce budworm disturbance is rarely stand replacing and is more likely to cause partial mortality events although mortality can continue to occur after the outbreak has ended due to weakening of live trees [48]. The severity of the outbreak varies depending on the availability and density of mature host species [36]. Data are available for one recorded outbreak in our case study region from 1972–1992 [41]. Based on field reports from similar regions [48], we estimated that tree mortality during this outbreak likely occurred after 3 to 6 consecutive years of defoliation. Since there are only data for one outbreak in this area, estimates of the return interval and levels of mortality carry high uncertainty. Additionally, the defoliation patterns were likely influenced by disturbance legacies from other natural disturbance events as well as logging, and forest management practices are likely to alter the characteristics of future insect outbreaks in the region [49].

#### Step 1—vegetation dynamics

As the primary host of spruce budworm, balsam fir was the only disturbance-prone vegetation type used in this analysis. We did not include the secondary hosts due to the resilience of black spruce to defoliation [50] and the absence and rarity of red and white spruce in the case study region. Balsam fir trees have advance regeneration (i.e. a bank of seedlings under the canopy), and young trees are less vulnerable to outbreaks [36]. Therefore, for the purpose of this model, we considered balsam fir stands to be self-replacing following high-severity spruce budworm disturbance [36, 37]. We think this is an acceptable simplification in our study region, but other regions may need to account for more complex forest dynamics.

#### Step 2 –data

*Disturbance events*. We mapped spruce budworm disturbance using aerial survey data from 1972–1992 collected as part of the Forest Insect and Disease Survey (FIDS) by Natural Resources Canada [51]. This vector dataset contains yearly polygons of observed percent defoliation categorized as low (0% - 25%), moderate (26% - 75%) or high (76% - 100%) severity. The total footprint of the outbreak in our case study region was 55,039 km^2^, 75% of which was moderate to severe defoliation. MacLean and MacKinnon [52] found aerial surveys to be on average 82% accurate when compared to ground-based surveys between 1984 and 1993, although the earlier years were the least accurate with correct classifications falling as low as 66%. We excluded light severity defoliation from the analysis because it is the most difficult to correctly classify in aerial surveys, and defoliation less than 30% has been reported not to cause mortality [52]. After clipping the data to our study area and projecting (to NAD83 Albers), moderate to severe defoliation polygons in our dataset ranged in size from 0.015 km^2^ to 10,000 km^2^.

We defined disturbance events as contiguous polygons of *X* or more consecutive years of defoliation to create four different disturbance scenario maps where *X* is set to 3, 4, 5 or 6 years. Yearly outbreak polygons were combined to map the total area that experienced at least *X* consecutive years of defoliation at some point during the outbreak. This assumes mortality begins after *X* years of moderate to high severity defoliation. We repeat the entire analysis for each of these four disturbance scenarios. Hereafter we refer to the disturbance scenarios as e.g., *X* = 3, which indicates the area that had three or more consecutive years of defoliation at any time during the outbreak. An alternate method would be to use a cumulative defoliation approach by summing yearly defoliation percentages to get the cumulative current year defoliation for the outbreak [53]. Blais [54] for example linked a 75% mortality rate to an average cumulative defoliation value of 700% (the equivalent of 7 age classes of foliage). While this method may more accurately define disturbance polygons, an accurate estimate of cumulative defoliation in our case study is difficult given the coarseness of the severity classes in the aerial survey data.

Our disturbance maps are a coarse scale characterisation aimed at capturing the disturbance process. They are not intended to represent an area of full mortality, nor do we expect all balsam fir trees in the disturbed area to die. Trees surviving high-severity defoliation events can still display effects of disturbance such as reduction in growth volume, cessation of height growth, and top-kill [48, 55]. High severity defoliation can be reported in areas with relatively little balsam fir and vice versa. Due to the scale of the survey data, the disturbance polygons will include non-host species and non-forested areas captured during the aerial survey mapping.

*Balsam fir distribution*. To map vegetation, we used forest resource inventory data (hereafter forest inventory) [42]. The forest inventory includes information on species, percent cover, average stand age in 2012 reported in 20-year intervals (0–20, 21–40, 41–60, 61–80, 81–100, 101–120, and >121 years), and the years of any harvesting. The forest inventory covers 76% of the case study region, with data missing from some sparsely forested areas and National Parks. The forest inventory covers 97% of the spruce budworm defoliation footprint. We extracted all polygons where balsam fir made up 100% of the canopy. These polygons ranged in size from 0.001 km^2^ to 4.1 km^2^. They were projected to NAD83 Albers and converted to 100m resolution raster layers, for a minimum mapping unit of 0.01 km^2^. We only mapped balsam fir cells with 100% cover because vulnerability to spruce budworm increases with balsam fir content [36, 53, 56]. Balsam fir mortality tends to be lower in the presence of hardwood species, likely due to an increase in numbers or diversity of natural budworm enemies, or greater losses of dispersing larvae [40, 56–58].

#### Step 3—initial and dynamic conditions

Having collated data and defined the vegetation dynamics, we next defined a set of initial and dynamic conditions to be met by MDRs. These conditions aim to identify MDRs that capture the spruce budworm disturbance process and maintain resilience to the disturbance by representing a range of balsam fir age classes over time. Initial conditions are defined by the largest observed spruce budworm disturbance event (i.e. maximum event size) and representation of disturbance-prone vegetation types (i.e. balsam fir).

*Initial conditions*. For each ecoregion, we calculated the initial conditions (*M*_*SBW*_ and *y*_*SBW*_) that a spruce budworm MDR must satisfy. We use the symbols *M* and *y* to match the original MDR formulation of Leroux et al. [10]. *M*_*SBW*_ aims to capture the disturbance footprint and *y*_*SBW*_ aims to maintain an area of host species greater than the largest observed mortality event.


$$M_{SBW}=\text{maximum}\;\text{event}\;\text{size}$$ (Eq 1)

The maximum event size was the largest polygon from the ecoregions’ disturbance map, where disturbance was mapped as all areas with at least *X* years of consecutive defoliation during the 1972–1992 outbreak. We repeated the analysis with *X* values of 3, 4, 5 and 6. Any MDR must be at least as large as *M*_*SBW*_. Unlike Leroux et al.’s [10] estimates of the maximum fire size [59], statistical methods to predict the largest spruce budworm disturbance event based on historical data do not exist, so our estimate is limited to observations in the historic record. *M*_*SBW*_ is designed to capture the scale of the disturbance process and is not intended to represent an area of complete mortality. As mentioned above, the mapped disturbance area is a coarse representation of the disturbance footprint and should capture a range of disturbance effects.

We also place an initial condition on balsam fir content to ensure a minimum representation of host species. This is a conservation target with the goal of maintaining a range of age classes in the MDR over time. At this stage, the balsam fir target can be met by any age class (we specifically test for the maintenance of age classes with the dynamic conditions described below). We set this target by estimating the largest host mortality event observed in the 1972–1992 outbreak. Any MDR must contain an area of balsam fir greater than *y*_*SBW*_:

$$y_{SBW}=max\left(bf_{1},\ldots ,bf_{n}\right)$$ (Eq 2)

where *bf* is the estimated area of balsam fir mortality within each of the *n* polygons making up the disturbance map for a given disturbance scenario (e.g. *X* = 3). We mapped the expected areas of mortality from the 1972–1992 outbreak using the 100m forest inventory grids to find balsam fir cells aged 20–40 years as of 2012; these stands originated during or immediately following the outbreak (i.e. 1972–1992). We removed any cells known to have been harvested during this period (4% of the mapped grid cells). In most cases the polygon representing the maximum event size (*M*_*SBW*_) contained the largest area of balsam fir mortality.

*Dynamic conditions*. Dynamic conditions were the same across ecoregions. For this analysis, an MDR was required to maintain three age classes of balsam fir at a minimum threshold of 1km^2^, representing young, mature, and old forests (1–40, 41–80, and >80 years). These age classes align with the 30-40-year interval observed between outbreaks in eastern Canada. Representation of age classes avoids a ‘resilience gap’ whereby, for example, a long-term loss of old-growth forest can occur if there are no mature stands to replace the old stands lost to disturbance. Resilience gaps can lead to long-term declines in biodiversity and increased uncertainty around ecosystem services [60]. In order to investigate the sensitivity of the results to the minimum threshold, we also ran the analysis using a minimum threshold set to the minimum mapping unit (0.01km^2^), and a larger threshold of 2km^2^.

#### Step 4—iterative MDR search

*Moving window for candidate MDRs*. We used a moving window analysis in ArcGIS 10.3 [61] to identify candidate MDRs that met the initial conditions of *M*_*SBW*_ and *y*_*SBW*_ for each disturbance scenario (*X* = 3, 4, 5, 6). The square moving window with an initial area of *M*_*SBW*_ was centered on each cell of the balsam fir grid and clipped to the ecoregion. For each window, the area of balsam fir inside the window was tested against the *y*_*SBW*_ target. All windows meeting the initial conditions of *M*_*SBW*_ and *y*_*SBW*_ were then tested against the dynamic conditions using an aspatial simulation model. Simulations were run for 250 years and repeated 100 times (following Leroux et al. [10]). If none of the candidate MDRs met the dynamic conditions, the width of the square window was increased by one cell and the analysis repeated. The area of a candidate MDR passing both the initial and dynamic conditions was the spruce budworm MDR for that ecoregion.

*Simulation model for dynamic conditions*. In order to test any candidate MDRs for the dynamic conditions described above, we developed a simulation model of vegetation dynamics following spruce budworm disturbance. The scale of analysis for each simulation was the ecoregion, and simulations were done for all four disturbance scenarios (*X* = 3, 4, 5, or 6).

Landscape disturbance and succession simulation models for defoliating insects lag behind those of fire [62]. In the absence of a spruce budworm module for Leroux et al.’s CONSERV model [10], a landscape simulator specifically designed for evaluating reserves, we developed an aspatial model to simulate changes in host species age class distributions within reserves and on the broader landscape through multiple outbreaks. Our simple model was initialized using species and age classes from the forest inventory grids. Each cell operates independently (i.e. there is no spread component to the outbreak) and mortality is assigned based on cell age and a mortality probability.

Age classes from the forest inventory [42] (0–20, 21–40, 41–60, 61–80, 81–100, 101–120, and >121 years) were assigned to each cell in the 100m grid of 100% balsam fir cells for each ecoregion. We may be underestimating the amount of balsam fir on the landscape since we are not accounting for mixed stands that could succeed to balsam fir. Due to the lower vulnerability of mixed stands [56], we do not expect this to have a large impact on our results, although in other systems where mixed stands are more common this should be accounted for.

During the simulation, age was updated for each cell at 20-year time steps. At each step, cells were aged into the next age class, up to a maximum class of >121 where small-scale gap dynamics are assumed to occur [63].

We triggered outbreaks at 40-year intervals. For each outbreak, cells over the age of 60 were assigned to either survive or die based on a randomly selected mortality probability between 0.78 and 0.89. Dead cells were reset to age 0–20 years after a one timestep (20 year) delay designed to simulate the length of the outbreak and the fact that stands can take multiple years to die following defoliation [48]. We only allowed mortality for cells over 60 years of age because outbreak mortality disproportionately effects mature balsam fir trees [36]. The sample of mortality probabilities (0.78 to 0.89) was based on published estimates of balsam fir mortality in mature balsam fir stands in eastern Canada [36, 48]. For each simulation run, the area of balsam fir in age classes of 1–40, 41–80 and >80 was calculated at each time step within any candidate MDR reserves being tested. If the area of one or more balsam fir age classes fell below the minimum threshold of 1km^2^, the MDR failed. The simulation model and all statistical analyses were done in R version 3.6.1 [64].

**Fig 2:**
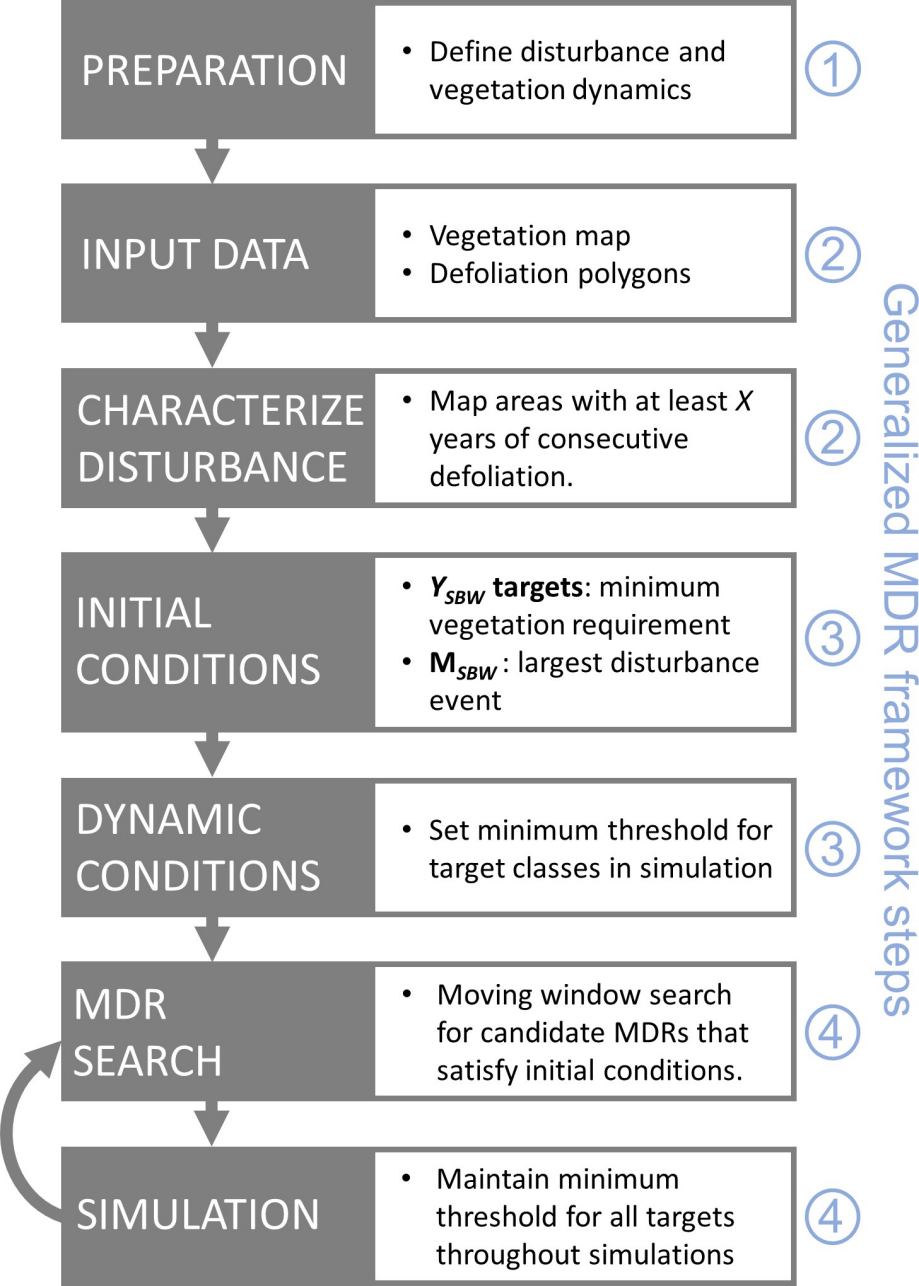
Generalized work flow for calculating spruce budworm MDR values. Analysis steps are shown in boxes, and the associated steps from the generalized MDR framework for defoliating insects are shown in blue (1: vegetation dynamics, 2: data, 3: initial and dynamic conditions, 4: iterative MDR search). Details can be found in the generalized MDR framework steps 1–4 and Eqs 1 and 2.

A workflow for the case study is demonstrated in Fig 2, along with the associated steps in the generalized MDR framework.

#### Evaluation of existing reserves

Using the MDR values identified for each ecoregion, we evaluated existing protected areas in our study region as an example of how our generalized MDR framework can be applied to regional conservation planning at the ecoregion scale. This is for demonstration purposes only and is not a complete conservation assessment of resilience to natural disturbance.

We used protected area polygons defined by the Conservation Area Reporting and Tracking System (CARTS) [65]. Protected areas sharing a border were combined into single contiguous areas and then clipped to the ecoregion for analysis. The resulting areas were compared to the MDR estimate for the ecoregion. For this example, we used the MDR value for *X* = 3, a minimum 3 years of consecutive defoliation. If the protected area size was greater than the ecoregion MDR, we also compared the area of the balsam fir within the protected area to the *y*_*SBW*_ area target for the ecoregion. If the *y*_*SBW*_ balsam fir target was met, the dynamic conditions of the protected area were tested using the simulation model. Simulations were only done for protected areas with full coverage of forest inventory.

## Results

### Ecoregion MDRs

For each ecoregion, we estimated a range of MDR values based on four disturbance scenarios defined by the minimum number of consecutive years of defoliation: *X* = 3, 4, 5, and 6 years (Table 1). Across ecoregions, the number of candidate reserves for each scenario ranged from 10 to over 1 million. The large number of options is due to the relatively small cell size of 0.01 km^2^ and because the moving window analysis evaluated windows centered on every grid cell, leading to candidate reserves with very high levels of overlap.

**Table 1:** Spruce budworm MDR results. Ecoregion area and total area of disturbance footprint are shown for each ecoregion and *X* scenario, along with *M*_*SBW*_, *y*_*SBW*_ and the final MDR value.

Ecoregion	Area, km^2^	Disturbance scenario (minimum consecutive years outbreak, X)	Total disturbed area, km^2^ (% of ecoregion)	Maximum event size, km^2^ (*M*_*SBW*_)	*y*_*SBW*_, km^2^	MDR, km^2^
1	28,728 km^2^	3	6905 (24.0)	1454	8.2	1454
		4	3422 (11.9)	450	6.3	450
		5	1363 (4.7)	135	2.4	135
		6	388 (1.4)	99	1.3	106 ^a^
2	37,904 km^2^	3	783.2 (2.1)	228	2.7	228
		4	285.6 (0.8)	137	2.0	184 ^a^
		5	93.9 (0.25)	25	0.7	184 ^a^
		6	-	-	-	-
3	16,589 km^2^	3	220.9 (1.3)	36	2.0	123 ^a^
		4	81.7 (0.5)	11	1.0	123 ^a^
		5	8.9 (0.05)	6	1.0	123 ^a^
		6	-	-	-	-
4	9,941 km^2^	3	2042.1 (20.6)	1058	41.5	1058
		4	613.7 (6.2)	163	13.3	163
		5	166.3 (1.7)	68	5.1	68
		6	-	-	-	-
5	8,549 km^2^	3	975.0 (11.6)	370	22.2	370
		4	605.1 (7.2)	304	18.7	304
		5	135.8 (1.6)	62	8.0	62
		6	12.9 (0.2)	6	1.5	50 ^a^
6	5,506 km^2^	3	1317.7 (29.1)	389	2.7	389
		4	553.9 (12.3)	125	1.2	292 ^a^
		5	205.0 (4.5)	88	0.8	292 ^a^
		6	-	-	-	-

“-“, Ecoregion does not have any areas with the indicated number of consecutive outbreak years.

^a^ MDR values had to be increased in order to meet the dynamic conditions.

**Fig 3:**
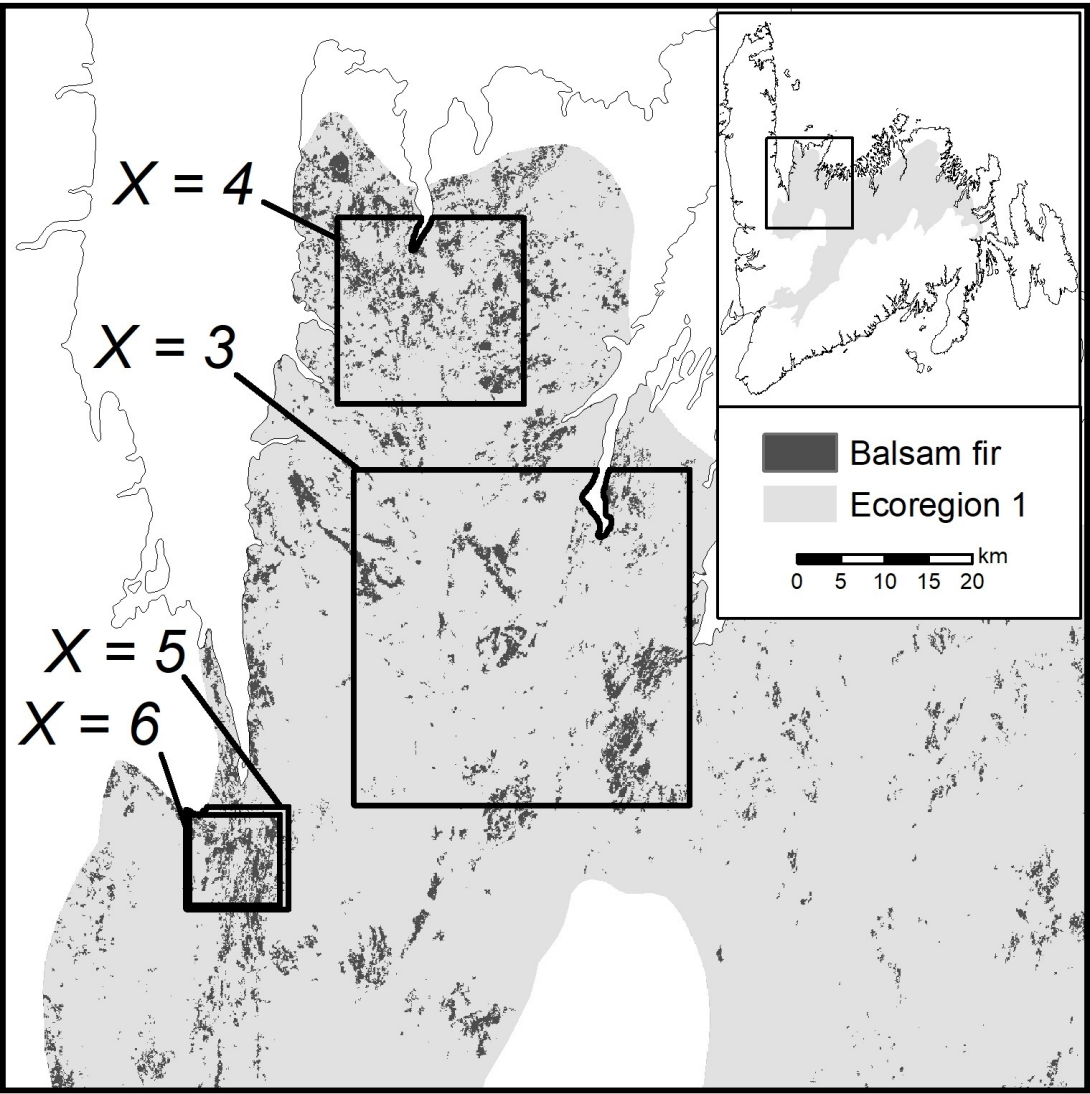
Candidate MDRs from ecoregion 1 that satisfied initial and dynamic conditions. Each MDR was estimated for a disturbance scenario defined by the minimum number of consecutive years of defoliation: *X* = 3, 4, 5, and 6 years. MDR values are in Table 1.

Across all ecoregions and disturbance scenarios, MDR estimates ranged from 50 km^2^ to 1454 km^2^ (Table 1 and Fig 3). Across all ecoregions, as *X* increases from 3 to 6 consecutive years of defoliation, the area of disturbance decreases, along with the largest disturbance event (*M*_*SBW*_) and the balsam fir target (*y*_*SBW*_). Within all ecoregions except ecoregion 3, the MDR estimate was inversely related to *X*, with a 1.2 to 15.6-fold difference between *X* = 3 and *X* = 5 or 6. There was a minimum reserve size needed to meet the dynamic conditions in ecoregion 3 which led to all disturbance scenarios having the same MDR size (Table 1). Across all ecoregions and scenarios, the maximum event size (*M*_*SBW*_) ranged from 6 km^2^ to 1454 km^2^ (Table 1). In all cases, the candidate MDR size tested for dynamic conditions was driven by *M*_*SBW*_ and not by the *y*_*SBW*_ balsam fir targets.

In 11 of the 20 scenarios, candidate MDRs with size *M*_*SBW*_ met the dynamic conditions as tested by the simulation model (i.e. MDR = *M*_*SBW*_ in Table 1). In nine cases, including all scenarios in ecoregion 3, candidate MDR sizes had to be increased by a factor of between 1.1 and 20.5 to meet the dynamic conditions (i.e. MDR > *M*_*SBW*_ in Table 1). Simulation results are shown in S1 Table. When using the minimum mapping unit (0.01km^2^) as the minimum threshold for age classes in the simulation, all candidate MDRs met the dynamic conditions (S2 Table). When testing a larger minimum threshold of 2km^2^, 15 of the 20 candidate MDRs had to be increased by a factor of 1.3 to 74.8 to meet the dynamic conditions (S3 Table).

### Evaluation of existing reserves

The protected area network in the case study region intersects with all ecoregions (S1 Fig). Of the six ecoregions, only ecoregions 2 and 3 had protected areas larger than the ecoregion MDR (n = 4, Table 2). In ecoregion 2, two protected areas exceeded the ecoregion MDR by 4.7- to 15-fold, and one met the *y*_*SBW*_ target. Due to incomplete forest inventory coverage, we were not able to assess *y*_*SBW*_ targets or test for dynamic conditions in these protected areas. In ecoregion 3, two protected areas exceeded the ecoregion MDR in size. We were not able to test *y*_*SBW*_ targets or dynamic conditions for Gros Morne National Park due to missing forest inventory. Little Grand Lake Wildlife Reserve/Little Grand Lake Provisional Ecological Reserve passed the initial conditions, and dynamic conditions were tested using the simulation model. This protected area had a heavily skewed age class distribution and failed to maintain all three age classes above the required 1 km^2^ threshold (S4 Table).

**Table 2:** Protected area attributes for protected areas larger than 100 km^2^ in the case study region. Protected areas sharing a border were combined and clipped to the ecoregion for analysis. MDR values for ecoregions are shown for reference. Balsam fir coverage and targets (*y*_*SBW*_*)* are only reported for protected areas with sizes larger than the ecoregion MDR.

Protected area name	Protected area size, km^2^	Intersecting Ecoregions	Area in ecoregion, km^2^	Spruce budworm MDR (*X* = 3), km^2^	Balsam fir in protected area, km^2^	*y*_*SBW*_, km^2^
Bay Du Nord Wilderness Reserve/Middle Ridge Wildlife Reserve	3522	1	33	1454	-	-
		**2**	**3489** ^a^	**228**	**0.2 (incomplete)**	**2.7**
Gros Morne National Park	1819	**3**	**993** ^a^	**123**	**Unknown**	**0.2**
		4	452	1058	-	-
		5	365	370	-	-
Little Grand Lake Wildlife Reserve/Little Grand Lake Provisional Ecological Reserve	1271	1	311	1454	-	-
		**3**	**872** ^a^	**123**	**56.7** ^b^	**2.0**
		4	88	1058	-	-
Avalon Wilderness Reserve/Salmonier Nature Park	1086	**2**	**1072** ^a^	**228**	**23.7 (incomplete)** ^b^	**2.7**
Terra Nova National Park/Terra Nova Migratory Bird Sanctuary	367	1	281	1454	-	-
		6	134	389	-	-
Main River Waterway Provincial Park/Main River Special Management Area	201	3	91	123	-	-
		5	110	370	-	-
Glover Island Public Reserve	178	4	178	1058	-	-

^a^ Area of reserve is greater than ecoregion MDR

^b^ Area of balsam fir in reserve is greater than *y*_*SBW*_

## Discussion

We extend the concept of minimum dynamic reserves (MDR) developed for wildfire [10] to spruce budworm outbreaks to illustrate conservation planning tools for regions where insect outbreaks are a primary natural disturbance. Here, MDRs are reserves large enough to capture the extent of the disturbance and maintain representation of host species age classes over time under active natural disturbance regimes. Methods for MDRs are a hybrid approach combining statistical and geospatial analysis with dynamic modelling and were originally developed for boreal ecosystems and a disturbance regime of stand-replacing fire. We demonstrate our spruce budworm method using a case study in the Canadian boreal forest. Overall, we found considerable variability across the study area in the size of MDRs estimated for spruce budworm. We attribute this variability to the unique patterns of host species and the size and severity of spruce budworm disturbance across ecoregions. We surmise that the methods we outline here hold promise for identifying large protected areas (i.e. mega-reserves [66]) for pro-active conservation planning in the world’s remaining intact regions [20, 21] as well as for informing reactive conservation and restoration efforts in lower intactness areas of biodiversity concern.

### Spruce budworm MDR case study

The primary driver of spruce budworm MDR size was the size of the largest disturbance event (*M*_*SBW*_). Reserves of this size always satisfied the vegetation targets we imposed via the initial conditions (Table 1). This is not surprising given that we only used a single disturbance-prone vegetation type. The ability of candidate MDRs to meet our dynamic conditions was limited in some ecoregions. In nine of the 20 MDR scenarios, failure to satisfy the dynamic conditions drove an increase in MDR size over the initial estimate. In all other cases, spatial coverage and configurations of host species age classes were sufficient to meet our dynamic conditions within a reserve the size of the largest disturbance event. The results are sensitive to the minimum threshold set for host species age classes in the simulation model. This is expected and highlights the importance of tying minimum thresholds to robust conservation targets. When using a smaller minimum threshold of 0.01km^2^ we found that none of the 20 scenarios required the initial MDR value to be increased (S2 Table), whereas a larger threshold of 2 km^2^ required 15 (of 20) scenarios to be increased. We used a minimum threshold aimed at maintaining representation of forest age classes. For conservation planning purposes, MDR options can be further constrained by tying minimum thresholds to specific conservation goals such as habitat requirements for focal species (e.g. [67]).

Our analysis is a first step towards incorporating budworm-defoliator dynamics into reserve design. We only considered a single host species and expect the addition of secondary host species to constrain the availability of candidate MDRs in our study region. Systems with multiple primary host species could be accommodated by including more vegetation classes and more complex vegetation dynamics. For example, ecosystems where spruce budworm causes significant damage to multiple hosts (e.g. [68]) would require MDRs to meet initial and dynamic conditions for each host species. Future work could build on our framework by incorporating more detailed vegetation and disturbance data, and spatial simulation models with multiple disturbance agents. Since spruce budworm and fire disturbances have been shown to interact at the landscape scale [69], simulation models accounting for interacting disturbances will refine MDR estimates in areas with overlapping disturbances. Incorporating multiple disturbances is an ongoing challenge in landscape simulation models [12, 70], but some examples exist [71, 72] including the Landis II model which has been parameterized for many landscapes and disturbance types [72, 73].

Our method is of primary interest in the eastern boreal where spruce budworm is a major disturbance, but the generalized MDR framework could be applied in other regions where insect disturbances are severe. Mountain pine beetle (*Dendroctonus ponderosae*) could be a good candidate, especially since the relationship between insect attack and tree mortality is less complex than for spruce budworm [74]. We did not consider climate change in our case study, but the MDR framework could test for resilience to future disturbance events by introducing projected disturbance regimes into the simulation models. This could be an important advancement to the framework since climate change is expected to alter both insect [75] and fire [11] disturbance regimes, and interactions between disturbances and climate warming could reduce ecosystem resilience and lead to unexpected ecosystem responses [32, 33].

### Protected areas evaluation

There is a growing recognition of the need for conservation planning to focus on ecological processes, rather than basing conservation decisions on a static view of biodiversity [2]. A number of studies have evaluated the long-term adequacy of reserve networks based on ecological processes such as fire [4, 10, 76] and flooding [5], and an increasing array of tools are available for incorporating processes such as natural disturbance into systematic conservation planning (see review in Leroux and Rayfield [3]). Several recent studies have evaluated the effectiveness of existing protected areas for protecting biodiversity [77–80], and Todd et al. [67] and Leroux et al. [4] simulated the effect of fire on habitat availability to evaluate reserve networks designed for species at risk. The MDR approach differs in that it estimates the area required for a single reserve to maintain representation of forest types under natural disturbance, thus allowing for the persistence of natural disturbance. MDR analyses are typically focused on estimating an area requirement for new reserves. The MDR framework can however be used to evaluate existing reserves for their ability to maintain ecological processes over time under natural disturbance. This can be done by comparing regional MDR values to existing reserve areas, and by testing existing reserves for dynamic conditions using simulation models. Our evaluation of existing protected areas within the study region was limited to areas with full forest inventory coverage, however in those regions, no protected areas met both the MDR size requirements and the dynamic conditions. The one protected area that met the initial conditions and could be tested with the simulation model was seven times larger than its ecoregion MDR but failed to meet the dynamic conditions due to the distribution of host species age classes. This indicates that size alone is not a sufficient measure of MDR status.

MDR estimates can be a useful tool to evaluate the risk to proposed or existing reserves in regions with active disturbance regimes. Reserves smaller than the regional MDR value for example could be at risk of losing important habitat for species of conservation concern. An example of this is the Northwest Forest Plan (NWFP) in the Pacific Northwest of the United States which sought to reduce population decline of the Northern Spotted Owl (*Strix occidentalis caurina*). The plan reduced forest harvesting in old growth forest habitat by setting aside Late Successional Reserves, starting in 1994 [81]. Reductions in habitat loss due to harvest have been offset by habitat loss due to increasing amounts of stand-replacing fire [82]. Bird populations associated with old-growth habitat have therefore continued to decline in the region [83]. In contrast to proactive planning approaches for maintaining ecological processes, this is an example of a region where the MDR could be applied to evaluate the risk of losing specific habitat types across a network of existing protected areas based on an active, and potentially shifting, disturbance regime.

We acknowledge that in some systems, MDR values may be so large that they are not operationally realistic, or very small due to low levels of natural disturbance. In these cases adaptations to the MDR goals may be needed, or different planning approaches such as dynamic reserves [76, 84, 85] could be explored.

### Limitations and future work

A limitation in the widespread application of these methods in conservation planning is the availability of accurate data. The prediction of mortality could be improved using a cumulative defoliation approach [53] which would require more accurate aerial survey polygons at the scale of our analysis. The relatively wide severity classes in the aerial survey data allow for broad classifications of moderate to severe defoliation, but do not facilitate exact percent calculations of cumulative mortality over multiple years. Higher quality defoliation data would also allow for more accurate disturbance maps. Improvements in remote sensing products such as the potential to use high-resolution spaceborne LiDAR (e.g., GEDI [86]) will allow for finer-resolution mapping and modelling of vegetation, reducing some of the uncertainty associated with current forest inventory maps.

Our simulation model was a simple representation of coupled spruce budworm disturbance and vegetation succession. The biggest limitation was a lack of any spatial spread in the spruce budworm outbreak. Development of a spatially explicit spruce budworm model that can evaluate candidate MDRs would allow for a more realistic simulation. This would also be a step towards an integrated landscape model that could consider all interacting disturbances.

Estimating the size of the largest potential disturbance event in a region becomes more accurate as the sample size of past events increases. Recent progress in paleolimnology is providing data on budworm outbreak dynamics and sizes several centuries into the past [87]. These data may improve our understanding of outbreak history with implications for conservation planning. Given that our analysis is only based on data from one outbreak, it is also possible that there are large areas of old growth that were not impacted by the last outbreak and may therefore be vulnerable to future outbreaks. In these cases, we could have underestimated the size of disturbance polygons and therefore MDR values. The testing of dynamic conditions using simulation models can partly compensate for this. For example, in our analysis of protected areas the Little Grand Lake protected area complex in ecoregion 3 met the MDR size and vegetation targets but failed the dynamic conditions due to a heavily skewed age class distribution. This is an important reminder that the MDR framework provides an estimate of the *minimum* reserve size required by searching the ecoregion for an optimal solution. A realized MDR in the ecoregion may need to be larger depending on its location.

## Conclusions

In this paper, we presented a generalized framework for estimating minimum dynamic reserves (MDRs) for spruce budworm. By doing so, we hope to stimulate further development of methods for conservation planning for diverse natural disturbances. Globally, there are relatively few very large terrestrial protected areas in forest ecosystems [88], and few remaining regions where large protected areas could be established [20, 21]. These regions are often characterized by active disturbance regimes that can produce large disturbance events such as insect outbreaks and fires in the boreal forest [16], blowdown in the Amazon rainforest [89], and hurricanes in coastal systems [9]. These processes should be incorporated into conservation planning to ensure the long-term persistence of biodiversity in these regions. Given recent calls for expansion of large protected areas in the world’s remaining intact regions [19, 21, 66, 90, 91], we believe the development of methods for i) evaluating the ability of existing protected areas to serve as MDRs and ii) identifying *de novo* protected areas that incorporate regional disturbances, is a significant step towards effective proactive conservation planning.

## Supporting information

S1 Fig Protected areas map. The protected area network used in the analysis intersected with the ecoregions making up the study region.
(JPG)

S1 Table Spruce budworm-based MDR simulation results. Reserve size: the area of the MDR being tested. Initial area: the amount of each age class in the reserve. Minimum area: the lowest value recorded for each age class throughout all 100 simulations. Also shown are the mean and standard deviation across all 100 simulations. In order to pass the simulation evaluation, all three age classes had to be maintained above a 1km^2^ threshold.
(DOCX)

S2 Table Spruce budworm-based MDR simulation results using a 0.01km^2^ minimum threshold. Reserve size: the area of the MDR being tested. Initial area: the amount of each age class in the reserve. Minimum area: the lowest value recorded for each age class throughout all 100 simulations. Also shown are the mean and standard deviation across all 100 simulations. In order to pass the simulation evaluation, all three age classes had to be maintained above the minimum mapping unit of 0.01km^2^.
(DOCX)

S3 Table Spruce budworm-based MDR simulation results using a 2km^2^ minimum threshold. Reserve size: the area of the MDR being tested. Initial area: the amount of each age class in the reserve. Minimum area: the lowest value recorded for each age class throughout all 100 simulations. Also shown are the mean and standard deviation across all 100 simulations. In order to pass the simulation evaluation, all three age classes had to be maintained above a 2km^2^ threshold.
(DOCX)

S4 Table Protected area simulation results. Only protected areas that met the MDR size and *y*_*SBW*_ requirements, and had full coverage of forest inventory data were evaluated. Reserve size: the area of the MDR being tested. Initial area: the amount of each age class in the reserve. Minimum area: the lowest value recorded for each age class throughout all 100 simulations. Also shown are the mean and standard deviation across all 100 simulations. In order to pass the simulation evaluation, all three age classes had to be maintained above a 1km^2^ threshold.
(DOCX)

## References

[1] Poiani KA, Richter BD, Anderson MG, Richter HE. Biodiversity conservation at multiple scales: functional sites, landscapes, and networks. Bioscience. 2000;50: 133–146. doi: 10.1641/0006-3568(2000)050[0133:BCAMSF]2.3.CO;2

[2] Pressey RL, Cabeza M, Watts ME, Cowling RM, Wilson KA. Conservation planning in a changing world. Trends Ecol Evol. 2007;22: 583–592. doi: 10.1016/j.tree.2007.10.001 17981360

[3] Leroux SJ, Rayfield B. Methods and tools for addressing natural disturbance dynamics in conservation planning for wilderness areas. Divers Distrib. 2014;20: 258–271. doi: 10.1111/ddi.12155

[4] Leroux SJ, Schmiegelow FKA, Cumming SG, Lessard RB, Nagy J. Accounting for system dynamics in rerserve design. Ecol Appl. 2007;17: 1954–1966. doi: 10.1890/06-1115.1 17974334

[5] Lourival R, Drechsler M, Watts ME, Game ET, Possingham HP. Planning for reserve adequacy in dynamic landscapes; maximizing future representation of vegetation communities under flood disturbance in the Pantanal wetland. Divers Distrib. 2011;17: 297–310. doi: 10.1111/j.1472-4642.2010.00722.x

[6] Drechsler M, Lourival R, Possingham HP. Conservation planning for successional landscapes. Ecol Modell. 2009;220: 438–450. doi: 10.1016/j.ecolmodel.2008.11.013

[7] Game ET, Watts ME, Wooldridge S, Possingham HP. Planning for persistence in marine reserves: A question of catastrophic importance. Ecol Appl. 2008;18: 670–680. doi: 10.1890/07-1027.1 18488626

[8] Pickett STA, Thompson JN. Patch dynamics and the design of nature reserves. Biol Conserv. 1978;13: 27–37. doi: 10.1016/0006-3207(78)90016-2

[9] Allison GW, Gaines SD, Lubchenco J, Possingham HP. Ensuring persistence of marine reserves: catastrophes require adopting an insurance factor. Ecol Appl. 2003;13: 8–24. doi: 10.1890/1051-0761(2003)013[0008:EPOMRC]2.0.CO;2

[10] Leroux SJ, Schmiegelow FKA, Lessard RB, Cumming SG. Minimum dynamic reserves: a framework for determining reserve size in ecosystems structured by large disturbances. Biol Conserv. 2007;138: 464–473. doi: 10.1016/j.biocon.2007.05.012

[11] Flannigan MD, Krawchuk MA, De Groot WJ, Wotton BM, Gowman LM. Implications of changing climate for global wildland fire. Int J Wildl Fire. 2009;18: 483–507. doi: 10.1071/WF08187

[12] Seidl R, Thom D, Kautz M, Martin-Benito D, Peltoniemi M, Vacchiano G, et al. Forest disturbances under climate change. Nat Clim Chang. 2017;7: 395–402. doi: 10.1038/nclimate3303 28861124 PMC5572641

[13] Angelstam P, Kuuluvainen T. Boreal forest disturbance regimes, successional dynamics and landscape structures: a European perspective. Ecol Bull. 2004; 117–136. Available: http://www.jstor.org/stable/20113303

[14] Weber MG, Stocks BJ. Forest fires and sustainability in the boreal forests of Canada. Ambio. 1998;27: 545–550.

[15] Bond WJ, Keeley JE. Fire as a global “herbivore”: The ecology and evolution of flammable ecosystems. Trends Ecol Evol. 2005;20: 387–394. doi: 10.1016/j.tree.2005.04.025 16701401

[16] Bergeron Y, Fenton NJ. Boreal forests of eastern Canada revisited: old growth, nonfire disturbances, forest succession, and biodiversity. Botany. 2012;90: 509–523. doi: 10.1139/b2012-034

[17] Brandt JP, Flannigan MD, Maynard DG, Thompson ID, Volney WJA. An introduction to Canada’s boreal zone: ecosystem processes, health, sustainability, and environmental issues. Environ Rev. 2013;21: 207–226. doi: 10.1139/er-2013-0040

[18] Andrew ME, Wulder MA, Coops NC. Identification of de facto protected areas in boreal Canada. Biol Conserv. 2012;146: 97–107. doi: 10.1016/j.biocon.2011.11.029

[19] Watson JEM, Evans T, Venter O, Williams B, Tulloch A, Stewart C, et al. The exceptional value of intact forest ecosystems. Nat Ecol Evol. 2018;2. doi: 10.1038/s41559-018-0490-x 29483681

[20] Watson JEM, Venter O, Lee J, Jone KR, Robinson JG, Possingham HP, et al. Protect the last of the wild. Nature. 2018;563: 27–30. doi: 10.1038/d41586-018-07183-6 30382225

[21] Watson JEM, Shanahan DF, Di Marco M, Allan J, Laurance WF, Sanderson EW, et al. Catastrophic declines in wilderness areas undermine global environment targets. Curr Biol. 2016;26: 2929–2934. doi: 10.1016/j.cub.2016.08.049 27618267

[22] Baker WL. The landscape ecology of large disturbances in the design and management of nature reserves. Landsc Ecol. 1992;7: 181–194. doi: 10.1007/BF00133309

[23] Wiersma YF, Beechey TJ, Oosenbrug BM, Meikle JC. Protected Areas in Northern Canada: designing for ecological integrity, Phase 1 Report. CCEA Occassional Paper No. 16. Ottawa, Ontario, Canada; 2005.

[24] Kneeshaw D, Gauthier S. Old growth in the boreal forest: a dynamic perspective at the stand and landscape level. Environ Rev. 2003;11: S99–S114. doi: 10.1139/a03-010

[25] Shugart HH, West DC. Long-term dynamics of forest ecosystems: computer simulation models, which allow for numerous seedlings and the long lives of large trees, predict how forests will respond to different management techniques. Am Sci. 1981;69: 647–652.

[26] Arcese P, Sinclair ARE. The role of protected areas as ecological baselines. J Wildl Manage. 1997;61: 587–602. doi: 10.2307/3802167

[27] Schmiegelow FKA, Cumming SG, Lisgo KA, Leroux SJ, Krawchuk MA. Catalyzing large landscape conservation in Canada’s boreal systems: the BEACONs Project experience. In: Lewitt JN, editor. Conservation Catalysts: The Academy as Nature’s Agent. Lincoln Institute of Land Policy, Cambridge Massachusetts; 2014. pp. 97–122.

[28] Wiersma YF. Environmental benchmarks vs. ecological benchmarks for assessment and monitoring in Canada: Is there a difference? Environ Monit Assess. 2005;100: 1–9. doi: 10.1007/s10661-005-7055-6 15727295

[29] Simard M, Romme W. Do mountain pine beetle outbreaks change the probability of active crown fire in lodgepole pine forests? Ecol Monogr. 2011;81: 3–24. doi: 10.1890/10-1176.1

[30] Buma B. Disturbance interactions: characterization, prediction, and the potential for cascading effects. 2015;6: 70. doi: 10.1890/ES15-00058.1

[31] Buma B, Wessman C. Disturbance interactions can impact resilience mechanisms of forests. Ecosphere. 2011;2. doi: 10.1890/ES11-00038.1

[32] Johnstone JF, Allen CD, Franklin JF, Frelich LE, Harvey BJ, Higuera PE, et al. Changing disturbance regimes, ecological memory, and forest resilience. Front Ecol Environ. 2016;14: 369–378. doi: 10.1002/fee.1311

[33] Turner MG, Calder WJ, Cumming GS, Hughes TP, Jentsch A, LaDeau SL, et al. Climate change, ecosystems and abrupt change: science priorities. Philos Trans R Soc B. 2020;375: 20190105. doi: 10.1098/rstb.2019.0105 31983326 PMC7017767

[34] De Grandpré L, Waldron K, Bouchard M, Gauthier S, Beaudet M, Ruel J-C, et al. Incorporating insect and wind disturbances in a natural disturbance-based management framework for the boreal forest. Forests. 2018;9: 471. doi: 10.3390/f9080471

[35] Kneeshaw D, Bergeron Y, Kuuluvainen T. Forest ecosystem structure and disturbance dynamics across the circumboreal forest. In: Millington A, Blumer M, Schickhoff U, editors. The SAGE handbook of Biogeography. SAGE Publications Ltd; 2011.

[36] MacLean DA. Vulnerability of fir-spruce stands during uncontrolled spruce budworm outbreaks: a review and discussion. For Chron. 1980;56: 213–221. doi: 10.5558/tfc56213-5

[37] Thompson ID, Larson DJ, Montevecchi WAM. Characterization of old “wet boreal” forests, with an example from balsam fir forests of western Newfoundland. Environ Rev. 2003;11: S23–S46. doi: 10.1139/a03-012

[38] MacLean DA. Effects of spruce budworm outbreaks on the productivity and stability of balsam fir forests. For Chron. 1984;60: 273–279. doi: 10.5558/tfc60273-5

[39] Morin H. Dynamics of balsam fir forests in relation to spruce budworm outbreaks in the Boreal Zone of Quebec. Can J For Res. 1994;24: 730–741. doi: 10.1139/x94-097

[40] Su Q, MacLean DA, Needham TD. The influence of hardwood content on balsam fir defoliation by spruce budworm. Can J For Res. 1996;26: 1620–1628. doi: 10.1139/x26-182

[41] Arsenault A, LeBlanc R, Earle E, Brooks D, Clarke B, Lavigne D, et al. Unravelling the past to manage Newfoundland’s forests for the future. For Chron. 2016;92: 487–502. doi: 10.5558/tfc2016-085

[42] Forest Inventory. Newfoundland and Labrador Department of Fisheries, Forestry and Agriculture; 2012.

[43] Hennigar CR, MacLean DA, Quiring DT, Kershaw JA. Differences in spruce budworm defoliation among balsam fir and white, red, and black spruce. For Sci. 2008;54: 158–166. doi: 10.1093/forestscience/54.2.158

[44] Royama T. Population dynamics of the spruce budworm Choristoneura Fumiferana. Ecol Monogr. 1984;54: 429–462. doi: 10.2307/1942595

[45] Boulanger Y, Arseneault D. Spruce budworm outbreaks in eastern Quebec over the last 450 years. Can J For Res. 2004;34: 1035–1043. doi: 10.1139/x03-269

[46] Belyea RM. Death and deteriation of balsam fir weakened by spruce budworm defoliation in Ontario. Part II. An assessment of the role of associated insect species in the death of severely weakened trees. J For. 1952;50: 729–738. doi: 10.1093/jof/50.10.729

[47] Greenbank DO. Host species and the spruce budworm. Mem Entomol Soc Canada. 1963;95: 219–223. doi: 10.4039/entm9531219-1

[48] Ostaff DP, MacLean DA. Spruce budworm populations, defoliation, and changes in stand condition during an uncontrolled spruce budworm outbreak on Cape Breton Island, Nova Scotia. Can J For Res. 1989;19: 1077–1086. doi: 10.1139/x89-164

[49] Johns RC, Bowden JJ, Carleton DR, Cooke BJ, Edwards S, Emilson EJS, et al. A Conceptual Framework for the Spruce Budworm Early Intervention Strategy: Can Outbreaks be Stopped? Forests. 2019;10. doi: 10.3390/f10100910

[50] Nealis VG, Régnière J. Insect-host relationships influencing disturbance by the spruce budworm in a boreal mixedwood forest. Can J For Res. 2004;34: 1870–1882. doi: 10.1139/x04-061

[51] Sterner TE, Davidson AG. Forest insect and disease conditions in Canada 1980. Ottawa; 1981.

[52] MacLean DA, MacKinnon WE. Accuracy of aerial sketch-mapping estimates of spruce budworm defoliation in New Brunswick. Can J For Res. 1996;26: 2099–2108. doi: 10.1139/x26-238

[53] MacLean DA, Ostaff DP. Patterns of balsam fir mortality caused by an uncontrolled spruce budworm outbreak. Can J For Res. 1989;19: 1087–1095. doi: 10.1139/x89-165

[54] Blais JR. The vulnerability of balsam fir to spruce budworm attack in Northwestern Ontario, with special reference to the physiological age of the tree. For Chron. 1958;34: 405–422. doi: 10.5558/tfc34405-4

[55] Krause C, Gionest F, Morin H, MacLean DA. Temporal relations between defoliation caused by spruce budworm (Choristoneura fumiferana Clem.) and growth of balsam fir (Abies balsamea (L.) Mill.). Dendrochronologia. 2003;21: 23–31. doi: 10.1078/1125-7865-00037

[56] Bergeron Y, Leduc A, Morin H, Joyal C. Balsam fir mortality following the last spruce budworm outbreak in northwestern Quebec. Can J For Res. 1995;25: 1375–1384. doi: 10.1139/x95-150

[57] Campbell EM, MacLean DA, Bergeron Y. The severity of budworm-caused growth reductions in balsam fir/spruce stands with the hardwood content of surrounding forest landscapes. For Sci. 2008;54: 195–205. doi: 10.1093/forestscience/54.2.195

[58] Zhang B, MacLean DA, Johns RC, Eveleigh ES. Effects of hardwood content on balsam fir defoliation during the building phase of a spruce budworm outbreak. Forests. 2018;9: 104–118. doi: 10.3390/f9090530

[59] Cumming SG. A parametric model of the fire-size distribution. Can J For Res. 2001;31: 1297–1303. doi: 10.1139/x01-032

[60] Bengtsson J, Angelstam P, Elmqvist T, Emanuelsson U, Folke C, Ihse M, et al. Reserves, resilience and dynamic landscapes. Ambio. 2003;32: 389–396. doi: 10.1579/0044-7447-32.6.389 14627367

[61] ESRI. ArcGIS 10.3. Redlands, CA: Environmental Systems Research Institute; 2014.

[62] Sturtevant BR, Cooke BJ, Kneeshaw DD, Maclean DA. Modelling insect disturbance across forested landscapes: insights from the spruce budworm. Simulation Modeling of Forest Landscape Disturbances. Springer International Publishing; 2015. doi: 10.1007/978-3-319-19809-5

[63] McCarthy JW, Weetman G. Age and size structure of gap-dynamic, old-growth boreal forest stands in Newfoundland. Silva Fenn. 2006;40: 209–230. doi: 10.14214/sf.339

[64] R Core Team. R: A language and environment for statistical computing. R Foundation for Statistical Computing, Vienna, Austria. URL https://www.R-project.org/. 2019.

[65] Canadian Council on Ecological Areas. Conservation Area Reporting and Tracking System. 2016. Available: http://www.ccea.org/carts/

[66] Peres CA. Why we need megareserves in Amazonia. Conserv Biol. 2005;19: 728–733. doi: 10.1111/j.1523-1739.2005.00691.x

[67] Todd CR, Lindenmayer DB, Stamation K, Acevado-Cattaneo S, Smith S, Lumsden LF. Assessing reserve effectiveness: Application to a threatened species in a dynamic fire prone forest landscape. Ecol Modell. 2016;338: 90–100. doi: 10.1016/j.ecolmodel.2016.07.021

[68] Bouchard M, Kneeshaw D, Bergeron Y. Forest dynamics after successive spruce budworm outbreaks in mixedwood forests. Ecology. 2006;87: 2319–2329. doi: 10.1890/0012-9658(2006)87[2319:fdassb]2.0.co;2 16995632

[69] Candau J, Fleming RA, Wang X. Ecoregional patterns of spruce budworm-wildfire interactions in Central Canada’s forests. Forests. 2018;9: 137. doi: 10.3390/f9030137

[70] Keane RE, McKenzie D, Falk DA, Smithwick EAH, Miller C, Kellogg LKB. Representing climate, disturbance, and vegetation interactions in landscape models. Ecol Modell. 2015;309–310: 33–47. doi: 10.1016/j.ecolmodel.2015.04.009

[71] James PMA, Fortin MJ, Sturtevant BR, Fall A, Kneeshaw D. Modelling spatial interactions among fire, spruce budworm, and logging in the boreal forest. Ecosystems. 2011;14: 60–75. doi: 10.1007/s10021-010-9395-5

[72] Sturtevant BR, Miranda BR, Shinneman DJ, Gustafson EJ, Wolter PT. Comparing modern and presettlement forest dynamics of a subboreal wilderness: Does spruce budworm enhance fire risk? Ecol Appl. 2012;22: 1278–1296. doi: 10.1890/11-0590.1 22827135

[73] Sturtevant BR, Scheller RM, Miranda BR, Shinneman D, Syphard A. Simulating dynamic and mixed-severity fire regimes: a process-based fire extension for LANDIS-II. Ecol Modell. 2009;220: 3380–3393. doi: 10.1016/j.ecolmodel.2009.07.030

[74] MacLean DA. Impacts of insect outbreaks on tree mortality, productivity, and stand development. Can Entomol. 2016;148: S138–S159. doi: 10.4039/tce.2015.24

[75] Price DT, Alfaro RI, Brown KJ, Flannigan MD, Fleming RA, Hogg EH, et al. Anticipating the consequences of climate change for Canada’s boreal forest ecosystems. Environ Rev. 2013;365: 322–365. doi: 10.1139/er-2013-0042

[76] Rayfield B, James PMA, Fall A, Fortin M-J. Comparing static versus dynamic protected areas in the Quebec boreal forest. Biol Conserv. 2008;141: 438–449. doi: 10.1016/j.biocon.2007.10.013

[77] Barnes M, Szabo JK, Morris WK, Possingham H. Evaluating protected area effectiveness using bird lists in the Australian Wet Tropics. Divers Distrib. 2015;21: 368–378. doi: 10.1111/ddi.12274

[78] Barnes MD, Craigie ID, Harrison LB, Geldmann J, Collen B, Whitmee S, et al. Wildlife population trends in protected areas predicted by national socio-economic metrics and body size. Nat Commun. 2016;7: 1–9. doi: 10.1038/ncomms12747 27582180 PMC5025815

[79] Craigie ID, Baillie JEM, Balmford A, Carbone C, Collen B, Green RE, et al. Large mammal population declines in Africa’s protected areas. Biol Conserv. 2010;143: 2221–2228. doi: 10.1016/j.biocon.2010.06.007

[80] Geldmann J, Barnes M, Coad L, Craigie ID, Hockings M, Burgess ND. Effectiveness of terrestrial protected areas in reducing habitat loss and population declines. Biol Conserv. 2013;161: 230–238. doi: 10.1016/j.biocon.2013.02.018

[81] Thomas JW, Franklin JF, Gordon J, Johnson KN. The Northwest Forest Plan: origins, components, implementation experience, and suggestions for change. Conserv Biol. 2006;20: 277–287. doi: 10.1111/j.1523-1739.2006.00385.x 16903089

[82] Healey SP, Cohen WB, Spies TA, Moeur M, Pflugmacher D, Whitley MG, et al. The relative impact of harvest and fire upon landscape-level dynamics of older forests: lessons from the Northwest Forest Plan. Ecosystems. 2008;11: 1106–1119. doi: 10.1007/s10021-008-9182-8

[83] Phalan BT, Northrup JM, Yang Z, Deal RL, Rousseau JS, Spies TA, et al. Impacts of the Northwest Forest Plan on forest composition and bird populations. Proc Natl Acad Sci. 2019;116: 3322–3327. doi: 10.1073/pnas.1813072116 30718406 PMC6386667

[84] Alagador D, Cerdeira JO, Araújo MB. Shifting protected areas: scheduling spatial priorities under climate change. J Appl Ecol. 2014;51: 703–713. doi: 10.1111/1365-2664.12230

[85] D’Aloia CC, Naujokaitis-Lewis I, Blackford C, Chu C, Curtis JMR, Darling E, et al. Coupled networks of permanent protected areas and dynamic conservation areas for biodiversity conservation under climate change. Front Ecol Environ. 2019;7: 1–8. doi: 10.3389/fevo.2019.00027

[86] Qi W, Dubayah O. Combining Tandem-X InSAR and simulated GEDI lidar observations for forest structure mapping. Remote Sens Environ. 2016;187: 253–266. doi: 10.1016/j.rse.2016.10.018

[87] Navarro L, Harvey A-É, Morin H. Lepidoptera wing scales: a new paleoecological indicator for reconstructing spruce budworm abundance. Can J For Res. 2018;48: 302–308. doi: 10.1139/cjfr-2017-0009

[88] Leroux SJ, Krawchuk MA, Schmiegelow F, Cumming SG, Lisgo K, Anderson LG, et al. Global protected areas and IUCN designations: do the categories match the conditions? Biol Conserv. 2010;143: 609–616. doi: 10.1016/j.biocon.2009.11.018

[89] Esprito-Santo FDB, Keller M, Braswell B, Nelson BW, Frolking S, Vicente G. Storm intensity and old-growth forest disturbances in the Amazon region. Geophys Res Lett. 2010;37: 1–6. doi: 10.1029/2010GL043146

[90] Betts MG, Wolf C, Ripple WJ, Phalan B, Millers KA, Duarte A, et al. Global forest loss disproportionately erodes biodiversity in intact landscapes. Nature. 2017;547: 441–444. doi: 10.1038/nature23285 28723892

[91] Allan JR, Kormos C, Jaeger T, Venter O, Bertzky B, Shi Y, et al. Gaps and opportunities for the World Heritage Convention to contribute to global wilderness conservation. Conserv Biol. 2018;32: 116–126. doi: 10.1111/cobi.12976 28664996

