# Supplementary figures and images for: Conservation planning integrating natural disturbances: Estimating minimum reserve sizes for an insect disturbance in the boreal forest of eastern Canada
Source: PLoS One. 2022 May 9;17(5):e0268236. doi: 10.1371/journal.pone.0268236 (PMC9084528; doi:10.1371/journal.pone.0268236)

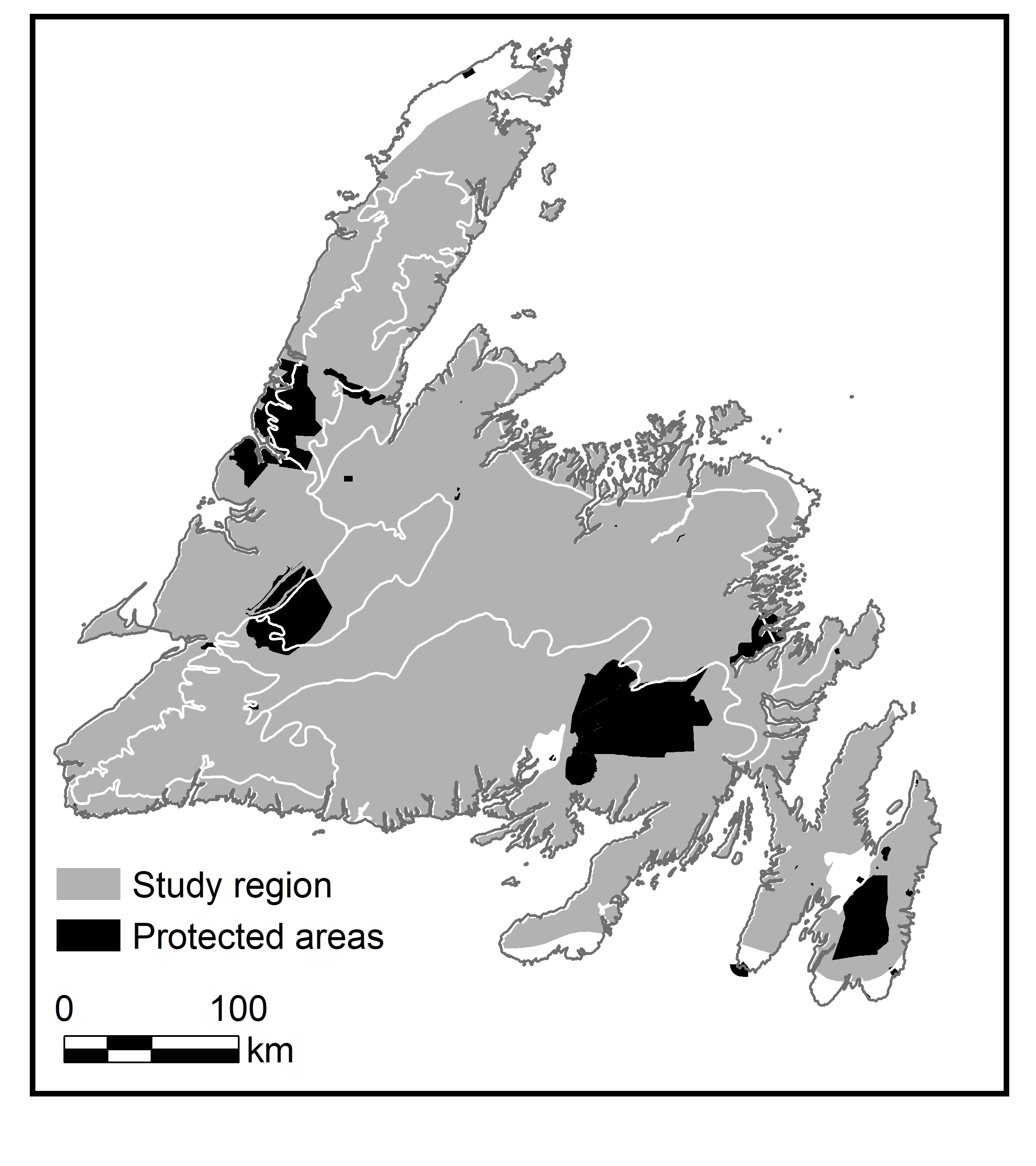

Supplement: S1 Fig — The protected area network used in the analysis intersected with the ecoregions making up the study region. (JPG) [file pone.0268236.s001.jpg]
