# Supplementary material for: Conservation planning integrating natural disturbances: Estimating minimum reserve sizes for an insect disturbance in the boreal forest of eastern Canada
Source: PLoS One. 2022 May 9;17(5):e0268236. doi: 10.1371/journal.pone.0268236 (PMC9084528; doi:10.1371/journal.pone.0268236)
Supplement: S3 Table — Reserve size: the area of the MDR being tested. Initial area: the amount of each age class in the reserve. Minimum area: the lowest value recorded for each age class throughout all 100 simulations. Also shown are the mean and standard deviation across all 100 simulations. In order to pass the simulation evaluation, all three age classes had to be maintained above a 2km2 threshold. (DOCX) [file pone.0268236.s004.docx]

| **Ecoregion** | **Outbreak length, years** | **Reserve size, km^2^** | **Area of balsam fir age class in reserve** | | | | |
| --- | --- | --- | --- | --- | --- | --- | --- |
|  |  |  | **Age class, years** | **Initial, km^2^** | **Minimum, km^2^** | **Mean, km^2^** | **St dev, km^2^** |
| 1 | 3 | 1454 | 0-40 | 82.52 | 21.46 | 56.17 | 22.94 |
|  |  |  | 40-80 | 6.21 | 6.21 | 34.71 | 19.29 |
|  |  |  | >80 | 23.76 | 3.85 | 21.61 | 19.76 |
|  | 4 | 450 | 0-40 | 51.29 | 20.76 | 44.2 | 15.03 |
|  |  |  | 40-80 | 20.49 | 20.49 | 28.9 | 8.26 |
|  |  |  | >80 | 19.2 | 3.65 | 17.88 | 13.94 |
|  | 5 | 174 | 0-40 | 20.68 | 11.26 | 24.49 | 8.57 |
|  |  |  | 40-80 | 10.28 | 9.95 | 15.6 | 3.94 |
|  |  |  | >80 | 19.88 | 2.01 | 10.75 | 8.16 |
|  | 6 | 174 | 0-40 | 20.68 | 11.31 | 24.49 | 8.57 |
|  |  |  | 40-80 | 10.28 | 9.95 | 15.61 | 3.95 |
|  |  |  | >80 | 19.88 | 2.02 | 10.74 | 8.16 |
| 2 | 3 | 480 | 0-40 | 16.67 | 11.4 | 22.6 | 7.6 |
|  |  |  | 40-80 | 21.95 | 10.6 | 16.12 | 3.08 |
|  |  |  | >80 | 9.6 | 2.02 | 9.51 | 6.94 |
|  | 4 | 480 | 0-40 | 16.67 | 11.39 | 22.6 | 7.6 |
|  |  |  | 40-80 | 21.95 | 10.6 | 16.12 | 3.09 |
|  |  |  | >80 | 9.6 | 2.01 | 9.5 | 6.95 |
|  | 5 | 480 | 0-40 | 16.67 | 11.45 | 22.6 | 7.6 |
|  |  |  | 40-80 | 21.95 | 10.6 | 16.12 | 3.09 |
|  |  |  | >80 | 9.6 | 2.03 | 9.51 | 6.94 |
| 3 | 3 | 434 | 0-40 | 16.61 | 11.39 | 37.27 | 19.39 |
|  |  |  | 40-80 | 6.97 | 4.57 | 22.45 | 15.32 |
|  |  |  | >80 | 54.61 | 2.03 | 18.47 | 17.88 |
|  | 4 | 434 | 0-40 | 16.61 | 11.19 | 37.27 | 19.37 |
|  |  |  | 40-80 | 6.97 | 4.57 | 22.44 | 15.3 |
|  |  |  | >80 | 54.61 | 2.02 | 18.49 | 17.86 |
|  | 5 | 434 | 0-40 | 16.61 | 11.14 | 37.28 | 19.4 |
|  |  |  | 40-80 | 6.97 | 4.57 | 22.44 | 15.34 |
|  |  |  | >80 | 54.61 | 2.01 | 18.47 | 17.88 |
| 4 | 3 | 1058 | 0-40 | 254.15 | 110.51 | 231.92 | 78.72 |
|  |  |  | 40-80 | 178.43 | 110.51 | 165.7 | 46.66 |
|  |  |  | >80 | 53.4 | 21.08 | 88.36 | 69.24 |
|  | 4 | 163 | 0-40 | 32.62 | 16.47 | 38.06 | 13.42 |
|  |  |  | 40-80 | 33.04 | 13.72 | 27.55 | 7.44 |
|  |  |  | >80 | 15.24 | 2.93 | 15.3 | 11.49 |
|  | 5 | 87 | 0-40 | 24.4 | 11.65 | 26.66 | 10.81 |
|  |  |  | 40-80 | 20.67 | 8.78 | 19.67 | 8.01 |
|  |  |  | >80 | 12.04 | 2.05 | 10.78 | 8.58 |
| 5 | 3 | 370 | 0-40 | 76.17 | 26.52 | 74.98 | 36.75 |
|  |  |  | 40-80 | 57.77 | 17.53 | 57.18 | 32.37 |
|  |  |  | >80 | 27.42 | 4.91 | 29.19 | 26.23 |
|  | 4 | 304 | 0-40 | 67.44 | 19.25 | 60.33 | 30.12 |
|  |  |  | 40-80 | 42.48 | 14.66 | 45.97 | 27.15 |
|  |  |  | >80 | 19.13 | 3.35 | 22.76 | 20.94 |
|  | 5 | 110 | 0-40 | 16.24 | 12.15 | 25.74 | 9.64 |
|  |  |  | 40-80 | 24.69 | 10.23 | 18.69 | 5.46 |
|  |  |  | >80 | 14.9 | 2.15 | 11.39 | 8.59 |
|  | 6 | 110 | 0-40 | 16.24 | 11.98 | 25.75 | 9.65 |
|  |  |  | 40-80 | 24.69 | 10.23 | 18.7 | 5.47 |
|  |  |  | >80 | 14.9 | 2.17 | 11.39 | 8.6 |
| 6 | 3 | 526 | 0-40 | 16.97 | 11.42 | 26.08 | 10.08 |
|  |  |  | 40-80 | 11.27 | 11.27 | 16.88 | 5.52 |
|  |  |  | >80 | 26.63 | 2.03 | 11.91 | 9.51 |
|  | 4 | 526 | 0-40 | 16.97 | 11.45 | 26.07 | 10.08 |
|  |  |  | 40-80 | 11.27 | 11.27 | 16.88 | 5.52 |
|  |  |  | >80 | 26.63 | 2.05 | 11.91 | 9.51 |
|  | 5 | 526 | 0-40 | 16.97 | 11.42 | 26.09 | 10.11 |
|  |  |  | 40-80 | 11.27 | 11.27 | 16.89 | 5.55 |
|  |  |  | >80 | 26.63 | 2.06 | 11.89 | 9.53 |
