# Supplementary material for: Conservation planning integrating natural disturbances: Estimating minimum reserve sizes for an insect disturbance in the boreal forest of eastern Canada
Source: PLoS One. 2022 May 9;17(5):e0268236. doi: 10.1371/journal.pone.0268236 (PMC9084528; doi:10.1371/journal.pone.0268236)
Supplement: S4 Table — Only protected areas that met the MDR size and ySBW requirements, and had full coverage of forest inventory data were evaluated. Reserve size: the area of the MDR being tested. Initial area: the amount of each age class in the reserve. Minimum area: the lowest value recorded for each age class throughout all 100 simulations. Also shown are the mean and standard deviation across all 100 simulations. In order to pass the simulation evaluation, all three age classes had to be maintained above a 1km2 threshold. (DOCX) [file pone.0268236.s005.docx]

| **Protected area** | **Ecoregion** | **Area of balsam fir age class in reserve** | | | | |
| --- | --- | --- | --- | --- | --- | --- |
|  |  | **Age class (years)** | **Initial, km^2^** | **Minimum, km^2^** | **Mean, km^2^** | **St dev, km^2^** |
| Little Grand Lake Wildlife Reserve/Little Grand Lake Provisional Ecological Reserve | 3 | 0-40 | 1.7 | 1.7 | 26.4 | 17.7 |
|  |  | 40-80 | 11.3 | 1.2 | 16.6 | 14.9 |
|  |  | >80 | 43.6 | 0.4 | 13.7 | 15.1 |
